# Supplementary material for: Cardiovascular risk among middle-aged Japanese adults with atopic dermatitis: A nested case–control study
Source: PLoS One. 2026 Jan 23;21(1):e0341337. doi: 10.1371/journal.pone.0341337 (PMC12829956; doi:10.1371/journal.pone.0341337)
Supplement: S1 Table — (DOCX) [file pone.0341337.s001.docx]

| **S1 Table. ICD-10 codes** |  |
| --- | --- |
| Atopic dermatitis | L20 |
| Ischemic heart disease | I20, I21, I22, I23, I24 |
| Cerebral hemorrhage | I60, I61, I62 |
| Cerebral infarction | I63 |
| Hypertension | I10, I11, I12, I13, I15 |
| Diabetes mellitus | E10, E11, E12, E13, E14 |
| Dyslipidemia | E78 |
| Hyperuricemia | E79 |
