# Supplementary material for: Cardiovascular risk among middle-aged Japanese adults with atopic dermatitis: A nested case–control study
Source: PLoS One. 2026 Jan 23;21(1):e0341337. doi: 10.1371/journal.pone.0341337 (PMC12829956; doi:10.1371/journal.pone.0341337)
Supplement: S2 Table — (DOCX) [file pone.0341337.s002.docx]

| **S2 Table. Drugs used to define the matching factors** | |
| --- | --- |
| Hypertension | Renin-angiotensin inhibitors, Calcium channel blockers, Beta-blockers, Alpha-blockers, Diuretics |
| Diabetes mellitus | SGLT2 inhibitors, DPP4 inhibitors, GLP1-receptor, Biguanides, Sulfonylureas, alpha-Glucosidase inhibitors, Thiazolidine, Glinides, Insulin |
|  |  |
| Dyslipidemia | Statins, Fibrates, Cholesterol Absorption Inhibitors, MTP inhibitors, PCSK9 Inhibitors, Probucol, Omega-3 Fatty Acids, Bile Acid Resins |
|  |  |
| Hyperuricemia | Xanthine Oxidase Inhibitors, Uricosuric Agents, Potassium citrate / Sodium citrate hydrate |
| Anticoagulant/antiplatelet agents | Antithrombotic agents (Tissue-type plasminogen activator (t-PA), Urokinase-type plasminogen activator, Batroxobin) |
|  |  |
|  | Antiplatelet agents (Aspirin, Ticlopidine, Ticagrelor, Clopidogrel, Prasugrel, Cilostazol, Rimabrost, Ozagrel, Sarpogrelate) |
|  |  |
|  | Anticoagulants (Heparin, Protamin sulfate, Dalteparin, Enoxaparin, Parnapalin, Danaparoid sodium, Argatroban, Warfarin, Prothrombin, Dabigatran, Idarucizumab, Edoxaban, Rivaroxaban, Apixaban, Andexanet) |
