# Supplementary material for: Cardiovascular risk among middle-aged Japanese adults with atopic dermatitis: A nested case–control study
Source: PLoS One. 2026 Jan 23;21(1):e0341337. doi: 10.1371/journal.pone.0341337 (PMC12829956; doi:10.1371/journal.pone.0341337)
Supplement: S3 Table — (DOCX) [file pone.0341337.s003.docx]

| **S3 Table. Case characteristics of IHD and stroke** |  |  |
| --- | --- | --- |
|  | IHD, n=1,247 | Stroke, n=1,563 |
| Age, median (IQR) | 54 [49-57] | 52 [48-56] |
| Sex, male, n (%) | 1033 (82.8) | 1044 (66.8) |
| Death, n (%) | 7 (0.6) | 57 (3.6) |
| Follow-up duration, median (IQR) | 60 [46-77] | 60 [46.5-78] |
| Presence of AD, n (%) | 31 (2.5) | 36 (2.3) |
| Presence of severe AD, n (%) |  |  |
| Prescription of class 1 TCS | 18 (1.4) | 11 (0.7) |
| Systematic treatment | 6 (0.5) | 8 (0.5) |
| Average monthly TCS dose among top 10% (g/month) | 38.9 | 27.7 |
| Hypertension, n (%) | 708 (56.8) | 767 (49.1) |
| Diabetes mellitus, n (%) | 379 (30.4) | 233 (14.9) |
| Dyslipidemia, n (%) | 652 (52.3) | 409 (26.2) |
| Hyperuricemia, n (%) | 194 (15.6) | 126 (8.1) |
| Anticoagulant/antiplatelet prescription, n (%) | 227 (18.2) | 163 (10.4) |
| Abbreviation: IQR; Interquartile range, IHD; ischemic heart diseases, AD; atopic dermatitis, TCS; topical corticosteroids | |  |
