# Supplementary material for: Cardiovascular risk among middle-aged Japanese adults with atopic dermatitis: A nested case–control study
Source: PLoS One. 2026 Jan 23;21(1):e0341337. doi: 10.1371/journal.pone.0341337 (PMC12829956; doi:10.1371/journal.pone.0341337)
Supplement: S4 Table — (DOCX) [file pone.0341337.s004.docx]

| **S4 Table. Characteristics of cases and matched controls in the Sensitivity Analysis** | | |  |
| --- | --- | --- | --- |
|  | Cases, n=2,220 | Controls, n=22,200 |  |
| Age, median (IQR) | 53 [49-57] | 53 [49-56] |  |
| Sex, male, n (%) | 1597 (71.9) | 15,970 (71.9) |  |
| Follow-up duration, median (IQR) | 61 [47-78] | 60 [46-77] |  |
| Number of practice months, median (IQR) | 31 [16-48] | 32 [18-49] |  |
| Hypertension, n (%) | 981 (44.2) | 9810 (44.2) |  |
| Diabetes mellitus, n (%) | 260 (11.7) | 2600 (11.7) |  |
| Dyslipidemia, n (%) | 661 (29.8) | 6610 (29.8) |  |
| Hyperuricemia, n (%) | 143 (6.4) | 1430 (6.4) |  |
| Anticoagulant/antiplatelet prescription, n (%) | 165 (7.4) | 1650 (7.4) |  |
| Abbreviation: IQR; interquartile range |  |  |  |
| Matching factors: age (±1 years), sex, index month, follow-up duration (±12 months), number of practice months (±10 months), hypertension, diabetes mellitus, dyslipidemia, hyperuricemia, anticoagulant/antiplatelet prescription | | |  |
|  |  |  |  |
|  |  |  |  |
