# Supplementary material for: Cardiovascular risk among middle-aged Japanese adults with atopic dermatitis: A nested case–control study
Source: PLoS One. 2026 Jan 23;21(1):e0341337. doi: 10.1371/journal.pone.0341337 (PMC12829956; doi:10.1371/journal.pone.0341337)
Supplement: S5 Table — (DOCX) [file pone.0341337.s005.docx]

| **S5-1 Table. Characteristics of cases with IHD and matched controls in the main analysis** | | |  |
| --- | --- | --- | --- |
|  | IHD, n=1,191 | Controls, n=11,910 |  |
| Matched factors |  |  |  |
| Age, median (IQR) | 54 [49-57] | 54 [49-57] |  |
| Sex, male, n (%) | 991 (83.2) | 9910 (83.2) |  |
| Hypertension, n (%) | 663 (55.7) | 6630 (55.7) |  |
| Diabetes mellitus, n (%) | 331 (27.8) | 3310 (27.8) |  |
| Dyslipidemia, n (%) | 614 (51.6) | 6140 (51.6) |  |
| Hyperuricemia, n (%) | 158 (13.3) | 1580 (13.3) |  |
| Anticoagulant/antiplatelet prescription, n (%) | 174 (14.6) | 1740 (14.6) |  |
| Unmatched factors |  |  |  |
| Follow-up duration, median (IQR) | 60 [46-78] | 60 [46-76] |  |
| Number of practice months, median (IQR) | 34 [20-51] | 59 [37-83] |  |
| Abbreviation: IQR; interquartile range |  |  |  |
| Matching factors: age, sex, index month, hypertension, diabetes mellitus, dyslipidemia, hyperuricemia, anticoagulant/antiplatelet prescription. | | |  |
|  |  |  |  |
