# Supplementary material for: Cardiovascular risk among middle-aged Japanese adults with atopic dermatitis: A nested case–control study
Source: PLoS One. 2026 Jan 23;21(1):e0341337. doi: 10.1371/journal.pone.0341337 (PMC12829956; doi:10.1371/journal.pone.0341337)
Supplement: S6 Table — (DOCX) [file pone.0341337.s006.docx]

| **S5-2 Table. Characteristics of cases with stroke and matched controls in the main analysis** | | |  |
| --- | --- | --- | --- |
|  | Stroke, n=1,532 | Controls, n=15,320 |  |
| Matched factors |  |  |  |
| Age, median (IQR) | 52 [48-56] | 52 [48-56] |  |
| Sex, male, n (%) | 1030 (67.2) | 10,300 (67.2) |  |
| Hypertension, n (%) | 743 (48.5) | 7,430 (48.5) |  |
| Diabetes mellitus, n (%) | 215 (14.0) | 2,150 (14.0) |  |
| Dyslipidemia, n (%) | 388 (25.3) | 3,880 (25.3) |  |
| Hyperuricemia, n (%) | 108 (7.0) | 1,080 (7.0) |  |
| Anticoagulant/antiplatelet prescription, n (%) | 140 (9.1) | 1,400 (9.1) |  |
| Unmatched factors |  |  |  |
| Follow-up duration, median (IQR) | 61 [46-78] | 61 [48-78] |  |
| Number of practice months, median (IQR) | 26 [13-43] | 52 [32-77] |  |
| Abbreviation: IQR; interquartile range |  |  |  |
| Matching factors: age, sex, index month, hypertension, diabetes mellitus, dyslipidemia, hyperuricemia, anticoagulant/antiplatelet prescription. | | |  |
|  |  |  |  |
