# Supplementary material for: Cardiovascular risk among middle-aged Japanese adults with atopic dermatitis: A nested case–control study
Source: PLoS One. 2026 Jan 23;21(1):e0341337. doi: 10.1371/journal.pone.0341337 (PMC12829956; doi:10.1371/journal.pone.0341337)
Supplement: S7 Table — (DOCX) [file pone.0341337.s007.docx]

| **S6-1 Table. Comparison of AD characteristics between cases with IHD and matched controls in the main analysis** | | | |
| --- | --- | --- | --- |
|  | Cases, n=1,191 | Controls, n=11,910 | OR (95% CIs) |
| Prevalence of AD, n (%) | 31 (2.6) | 316 (2.7) | 0.98 (0.66-1.40) |
| Prevalence of severe AD, n (%) |  |  |  |
| Prescription for the top 10% of average monthly TCS doses (52.9 g/month) |  |  |  |
| Yes (severe) | 0 (0) | 34 (0.3) | NA |
| No (mild) | 31 (2.6) | 282 (2.4) | 1.10 (0.74-1.57) |
| Use of Class 1 TCS |  |  |  |
| Yes (severe) | 18 (1.5) | 167 (1.4) | 1.08 (0.64-1.71) |
| No (mild) | 13 (1.1) | 149 (1.3) | 0.87 (0.47-1.48) |
| Systematic treatment |  |  |  |
| Yes (severe) | 6 (0.5) | 72 (0.6) | 0.83 (0.32-1.77) |
| No (mild) | 25 (2.1) | 244 (2.0) | 1.02 (0.66-1.52) |
| Content of systemic treatment |  |  |  |
| Oral corticosteroid | 5 (0.4) | 59 (0.5) |  |
| Calcineurin inhibitors | 2 (0.2) | 12 (0.1) |  |
| Dupilumab | 0 | 13 (0.1) |  |
| Baricitinib | 0 | 0 |  |

| **S6-1 Table. Comparison of AD characteristics between cases with IHD and matched controls in the main analysis (Continued)** | | | |
| --- | --- | --- | --- |
|  | Cases, n=1,191 | Controls, n=11,910 | p value |
| TCS, monthly average, g, median (IQR) | 10.6 [3.3-18.1] | 13.0 [4.5-30.2] | 0.33 |
| Top 10% for average monthly TCS dose, g | 38.9 | 54.8 |  |
| Follow-up duration of AD, median (IQR) | 51 [44.5-63.5] | 62.5 [43-80] | ＜0.05 |
| Number of practice months of AD, median (IQR) | 15 [9.5-25.5] | 22 [10.8-38] | ＜0.05 |
| Abbreviation: OR; odds ratio, IQR; interquartile range, AD; atopic dermatitis, TCS; topical corticosteroids | |  |  |
