# Supplementary material for: Cardiovascular risk among middle-aged Japanese adults with atopic dermatitis: A nested case–control study
Source: PLoS One. 2026 Jan 23;21(1):e0341337. doi: 10.1371/journal.pone.0341337 (PMC12829956; doi:10.1371/journal.pone.0341337)
Supplement: S8 Table — (DOCX) [file pone.0341337.s008.docx]

| **S6-2 Table. Comparison of AD characteristics between cases with stroke and matched controls in the main analysis** | | | |
| --- | --- | --- | --- |
|  | Stroke, n=1,532 | Controls, n=15,320 | OR (95% CIs) |
| Prevalence of AD, n (%) | 36 (2.3) | 423 (2.8) | 0.85 (0.59-1.18) |
| Prevalence of severe AD, n (%) |  |  |  |
| Prescription for the top 10% of average monthly TCS dose (40.7 g/month) |  |  |  |
| Yes (severe) | 1 (0.1) | 44 (0.3) | 0.38 (0.02-1.80) |
| No (mild) | 35 (2.3) | 379 (2.5) | 0.88 (0.61-1.23) |
| Use of Class 1 TCS |  |  |  |
| Yes (severe) | 11 (0.7) | 199 (1.3) | 0.55 (0.28-0.96) |
| No (mild) | 25 (1.6) | 224 (1.5) | 1.11 (0.71-1.65) |
| Systematic treatment |  |  |  |
| Yes (severe) | 8 (0.5) | 76 (0.5) | 1.05 (0.47-2.04) |
| No (mild) | 28 (1.8) | 347 (2.3) | 0.80 (0.53-1.16) |
| Content of systemic treatment |  |  |  |
| Oral corticosteroid | 8 (0.5) | 63 (0.4) |  |
| Calcineurin inhibitors | 0 | 10 (0.07) |  |
| Dupilumab | 0 | 10 (0.07) |  |
| Baricitinib | 0 | 1 (0.007) |  |
|  | 0 | 1 (0.007) |  |

| **S6-2 Table. Comparison of AD characteristics between cases with stroke and matched controls in the main analysis (Continued)** | | | |
| --- | --- | --- | --- |
|  | Cases, n=1,532 | Controls, n=15,320 | p value |
| TCS, monthly average, g, median (IQR) | 6.5[2.4-16.0] | 8.6[2.9-21.2] | 0.22 |
| Top 10% for average monthly TCS dose, g | 27.6 | 41.6 |  |
| Follow-up duration of AD, median (IQR) | 65.5[43.2-77.8] | 59[44-76] | 0.69 |
| Number of practice months of AD, median (IQR) | 19.5[8-33.2] | 18[8-35.5] | 0.99 |
| Abbreviation: OR; odds ratio, IQR; interquartile range, AD; atopic dermatitis, TCS; topical corticosteroids | |  |  |
