# Supplementary material for: Cardiovascular risk among middle-aged Japanese adults with atopic dermatitis: A nested case–control study
Source: PLoS One. 2026 Jan 23;21(1):e0341337. doi: 10.1371/journal.pone.0341337 (PMC12829956; doi:10.1371/journal.pone.0341337)
Supplement: S9 Table — (DOCX) [file pone.0341337.s009.docx]

| **S7 Table. Comparison of AD characteristics between cases and matched controls in the sensitivity analysis** | | | |
| --- | --- | --- | --- |
|  | Cases, n=2,220 | Controls, n=22,200 | OR (95% CIs) |
| Prevalence of AD, n (%) | 57 (2.6) | 373 (1.7) | 1.54 (1.15-2.02) |
| Prevalence of severe AD, n (%) |  |  |  |
| Prescription for the top 10% of average monthly TCS dose (38.2 g/month) |  |  |  |
| Yes (severe) | 4 (0.2) | 39 (0.2) | 1.03 (0.31-2.57) |
| No (mild) | 53 (2.4) | 334 (1.5) | 1.60 (1.18-2.13) |
| Use of Class 1 TCS |  |  |  |
| Yes (severe) | 25 (1.1) | 159 (0.7) | 1.59 (1.01-2.38) |
| No (mild) | 32 (1.4) | 214 (1.0) | 1.51 (1.02-2.16) |
| Systematic treatment |  |  |  |
| Yes (severe) | 10 (0.5) | 59 (0.3) | 1.71 (0.82-3.20) |
| No (mild) | 47 (2.1) | 314 (1.4) | 1.51 (1.09-2.04) |
| Content of systemic treatment |  |  |  |
| Oral corticosteroid | 10 (0.5) | 55 (0.2) |  |
| Calcineurin inhibitors | 1 (0.05) | 1 (0.005) |  |
| Dupilumab | 0 | 6 (0.03) |  |
| Baricitinib | 0 | 0 |  |
| Upadacitinib | 0 | 1 (0.005) |  |

| **S7 Table. Comparison of AD characteristics between cases and matched controls in the sensitivity analysis (Continued)** | | | | | |  |
| --- | --- | --- | --- | --- | --- | --- |
|  | Cases, n=2,220 | Controls, n=22,200 | | P value | |  |
| TCS, monthly average, g, median (IQR) | 8.6 [3-17.1] | 8.0 [2.85-23.2] | | 0.95 | |  |
| Top 10% for average monthly TCS dose, g | 32.7 | 38.5 | |  | |  |
| Follow-up duration of AD, median (IQR) | 61 [44-70] | 63 [46-81] | | 0.08 | |  |
| Number of practice months of AD, median (IQR) | 16 [8-32] | 15 [7-27] | | 0.38 | |  |
| Abbreviation: OR; odds ratio, IQR; Interquartile range, AD; Atopic dermatitis, TCS; topical corticosteroids |  |  | |  | |  |
| Matching factors: age (±1 years), sex, index month, follow-up duration (±12 months), number of practice months (±10 months), hypertension, diabetes mellitus, dyslipidemia, hyperuricemia, anticoagulant/antiplatelet prescription | | | | | |  |
|  |  |  |  |  |  |  |
|  | | |  | |  |  |
