# Supplementary material for: Cardiovascular risk among middle-aged Japanese adults with atopic dermatitis: A nested case–control study
Source: PLoS One. 2026 Jan 23;21(1):e0341337. doi: 10.1371/journal.pone.0341337 (PMC12829956; doi:10.1371/journal.pone.0341337)
Supplement: S10 Table — (DOCX) [file pone.0341337.s010.docx]

| **S8-1 Table. Characteristics of cases with IHD and matched controls in the sensitivity analysis** | | |  |
| --- | --- | --- | --- |
|  | Cases, n=958 | Controls, n=9,580 |  |
| Age, median (IQR) | 54 [49-57] | 54 [49-57] |  |
| Sex, male, n (%) | 784 (81.8) | 7840 (81.8) |  |
| Follow-up duration, median (IQR) | 61 [47-77] | 60 [46-77] |  |
| Number of practice months, median (IQR) | 36 [21-53] | 37 [22-54] |  |
| Hypertension, n (%) | 460 (48.0) | 4600 (48.0) |  |
| Diabetes mellitus, n (%) | 178 (18.6) | 1780 (18.6) |  |
| Dyslipidemia, n (%) | 441 (46.0) | 4410 (46.0) |  |
| Hyperuricemia, n (%) | 89 (9.3) | 890 (9.3) |  |
| Anticoagulant/antiplatelet prescription, n (%) | 95 (9.9) | 950 (9.9) |  |
| Abbreviation: IQR; interquartile range, IHD; ischemic heart diseases |  |  |  |
| Matching factors: age (±1 years), sex, index month, follow-up duration (±12 months), number of practice months (±10 months), hypertension, diabetes mellitus, dyslipidemia, hyperuricemia, anticoagulant/antiplatelet prescription | | |  |
|  |  |  |  |
|  |  |  |  |
