# Supplementary material for: Cardiovascular risk among middle-aged Japanese adults with atopic dermatitis: A nested case–control study
Source: PLoS One. 2026 Jan 23;21(1):e0341337. doi: 10.1371/journal.pone.0341337 (PMC12829956; doi:10.1371/journal.pone.0341337)
Supplement: S11 Table — (DOCX) [file pone.0341337.s011.docx]

| **S8-2 Table. Characteristics of cases with stroke and matched controls in the sensitivity analysis** | | |  |
| --- | --- | --- | --- |
|  | Cases, n=1,297 | Controls, n=12,970 |  |
| Age, median (IQR) | 53 [48-56] | 52 [48-56] |  |
| Sex, male, n (%) | 843 (65.0) | 8430 (65.0) |  |
| Follow-up duration, median (IQR) | 61 [47-79] | 61 [47-78] |  |
| Number of practice months, median (IQR) | 27 [14-44] | 28.5 [16-46] |  |
| Hypertension, n (%) | 549 (42.3) | 5490 (42.3) |  |
| Diabetes mellitus, n (%) | 99 (7.6) | 990 (7.6) |  |
| Dyslipidemia, n (%) | 247 (19.0) | 2470 (19.0) |  |
| Hyperuricemia, n (%) | 60 (4.6) | 600 (4.6) |  |
| Anticoagulant/antiplatelet prescription, n (%) | 76 (5.9) | 760 (5.9) |  |
| Abbreviation: IQR; interquartile range |  |  |  |
| Matching factors: age (±1 years), sex, index month, follow-up duration (±12 months), number of practice months (±10 months), hypertension, diabetes mellitus, dyslipidemia, hyperuricemia, anticoagulant/antiplatelet prescription | | |  |
|  |  |  |  |
|  |  |  |  |
