# Supplementary material for: Cardiovascular risk among middle-aged Japanese adults with atopic dermatitis: A nested case–control study
Source: PLoS One. 2026 Jan 23;21(1):e0341337. doi: 10.1371/journal.pone.0341337 (PMC12829956; doi:10.1371/journal.pone.0341337)
Supplement: S12 Table — (DOCX) [file pone.0341337.s012.docx]

| **S9-1 Table. Comparison of AD characteristics between cases with IHD and matched controls in the sensitivity analysis** | | | |
| --- | --- | --- | --- |
|  | Cases, n=958 | Controls, n=9,580 | OR (95% CIs) |
| Prevalence of AD, n (%) | 27 (2.8) | 164 (1.7) | 1.67 (1.08-2.47) |
| Prevalence of severe AD, n (%) |  |  |  |
| Prescription for the top 10% of average monthly TCS dose (37.35 g/month) |  |  |  |
| Yes (severe) | 4 (0.4) | 15 (0.2) | 2.70 (0.77-7.45) |
| No (mild) | 23 (2.4) | 149 (1.6) | 1.56 (0.98-2.38) |
| Use of Class 1 TCS |  |  |  |
| Yes (severe) | 15 (1.6) | 75 (0.8) | 2.02 (1.11-3.43) |
| No (mild) | 12 (1.3) | 89 (0.9) | 1.36 (0.71-2.40) |
| Systematic treatment |  |  |  |
| Yes (severe) | 15 (1.6) | 75 (0.8) | 2.30 (0.77-5.62) |
| No (mild) | 12 (1.3) | 89 (0.9) | 1.57 (0.97-2.41) |
| Content of systemic treatment |  |  |  |
| Oral corticosteroid | 5 (0.5) | 11 (0.1) |  |
| Calcineurin inhibitors | 1 (0.1) | 0 |  |
| Dupilumab | 0 | 0 |  |
| Baricitinib | 0 | 0 |  |
| Upadacitinib | 0 | 0 |  |

| **S9-1 Table. Comparison of AD characteristics between cases with IHD and matched controls in the sensitivity analysis (Continued)** | | | |  |
| --- | --- | --- | --- | --- |
|  | Cases, n=958 | Controls, n=9,580 | P value |  |
| TCS, monthly average, g, median (IQR) | 10.6 [3.3-16.4] | 7.7 [3.0-22.1] | 0.56 |  |
| Top 10% for average monthly TCS dose, g | 44 | 37.3 |  |  |
| Follow-up duration of AD, median (IQR) | 51 [44.5-64.5] | 59 [43-78] | 0.19 |  |
| Number of practice months of AD, median (IQR) | 13 [9.5-25.5] | 13 [6-27.2] | 0.50 |  |
| Abbreviation: OR; odds ratio, IQR; interquartile range, AD; atopic dermatitis, TCS; topical corticosteroids |  |  |  |  |
| Matching factors: age (±1 years), sex, index month, follow-up duration (±12 months), number of practice months (±10 months), hypertension, diabetes mellitus, dyslipidemia, hyperuricemia, anticoagulant/antiplatelet prescription | | | |  |
|  |  |  |  |  |
