# Supplementary material for: Cardiovascular risk among middle-aged Japanese adults with atopic dermatitis: A nested case–control study
Source: PLoS One. 2026 Jan 23;21(1):e0341337. doi: 10.1371/journal.pone.0341337 (PMC12829956; doi:10.1371/journal.pone.0341337)
Supplement: S13 Table — (DOCX) [file pone.0341337.s013.docx]

| **S9-2 Table. Comparison of AD characteristics between cases with stroke and matched controls in the sensitivity analysis** | | | |
| --- | --- | --- | --- |
|  | Cases, n=1,297 | Controls, n=12,970 | OR (95% CIs) |
| Prevalence of AD, n (%) | 31 (2.4) | 269 (2.1) | 1.16 (0.78-1.66) |
| Prevalence of severe AD, n (%) |  |  |  |
| Prescription for the top 10% of average monthly TCS dose (35.47 g/month) |  |  |  |
| Yes (severe) | 1 (0.1) | 29 (0.2) | 1.25 (0.84-1.81) |
| No (mild) | 30 (2.3) | 240 (1.9) | 1.56 (0.98-2.38) |
| Use of Class 1 TCS |  |  |  |
| Yes (severe) | 11 (0.8) | 107 (0.8) | 1.03 (0.52-1.84) |
| No (mild) | 20 (1.5) | 162 (1.2) | 1.24 (0.75-1.93) |
| Systematic treatment |  |  |  |
| Yes (severe) | 5 (0.4) | 41 (0.3) | 1.22 (0.42-2.82) |
| No (mild) | 26 (2.0) | 228 (1.8) | 1.14 (0.74-1.69) |
| Content of systemic treatment |  |  |  |
| Oral corticosteroid | 5 (0.4) | 37 (0.3) |  |
| Calcineurin inhibitors | 0 | 3 (0.02) |  |
| Dupilumab | 0 | 3 (0.02) |  |
| Baricitinib | 0 | 0 |  |
| Upadacitinib | 0 | 0 |  |

| **S9-2 Table. Comparison of AD characteristics between cases with stroke and matched controls in the sensitivity analysis (Continued)** | | | |  |
| --- | --- | --- | --- | --- |
|  | Cases, n=1,297 | Controls, n=12,970 | P value |  |
| TCS, monthly average, g, median (IQR) | 6.6 [2.2-18.3] | 6.7 [2.1-18.0] | 0.8 |  |
| Top 10% for average monthly TCS dose, g | 27.7 | 36.2 |  |  |
| Follow-up duration of AD, median (IQR) | 66 [41.5-76] | 59 [42-79] | 0.89 |  |
| Number of practice months of AD, median (IQR) | 21 [8-34] | 13 [6-26] | 0.11 |  |
| Abbreviation: OR; odds ratio, IQR; interquartile range, AD; atopic dermatitis, TCS; topical corticosteroids |  |  |  |  |
| Matching factors: age (±1 years), sex, index month, follow-up duration (±12 months), number of practice months (±10 months), hypertension, diabetes mellitus, dyslipidemia, hyperuricemia, anticoagulant/antiplatelet prescription | | | |  |
|  |  |  |  |  |
