# Supplementary material for: Cardiovascular risk among middle-aged Japanese adults with atopic dermatitis: A nested case–control study
Source: PLoS One. 2026 Jan 23;21(1):e0341337. doi: 10.1371/journal.pone.0341337 (PMC12829956; doi:10.1371/journal.pone.0341337)
Supplement: S14 Table — (DOCX) [file pone.0341337.s014.docx]

| **S10 Table. Characteristics of AD among the excluded population** | |
| --- | --- |
|  | Excluded, n=101,614 |
| Prevalence of AD, n(%) | 1917 (1.9%) |
| Prevalence of severe AD, n(%) |  |
| Prescription for the top 10% of average monthly TCS dose (38.2 g/month) |  |
| Yes (severe) | 215 (0.2) |
| No (mild) | 1702 (1.7) |
| Use of Class 1 TCS |  |
| Yes (severe) | 898 (0.9) |
| No (mild) | 1019 (1.0) |
| Systematic treatment |  |
| Yes (severe) | 379 (0.4) |
| No (mild) | 1538 (1.5) |
| Content of systemic treatment |  |
| Oral corticosteroid | 340 (0.3) |
| Calcineurin inhibitors | 28 (0.03) |
| Dupilumab | 44 (0.04) |
| Baricitinib | 3 (0.003) |
| Upadacitinib | 0 |
| Abbreviation: AD; atopic dermatitis, TCS; topical corticosteroids |  |
