# Supplementary material for: Cardiovascular risk among middle-aged Japanese adults with atopic dermatitis: A nested case–control study
Source: PLoS One. 2026 Jan 23;21(1):e0341337. doi: 10.1371/journal.pone.0341337 (PMC12829956; doi:10.1371/journal.pone.0341337)
Supplement: Attachment — Submitted filename: Response_to_Reviewers_auresp_2.docx [file pone.0341337.s016.docx]

Rebuttal letter

January 05, 2026

Seyed Aria Nejadghaderi

Academic Editor

PLOS ONE

Dear Editor,

We have revised our manuscript in accordance with the detailed suggestions you have graciously provided.

**Response to Editor**

1. If the reviewer comments include a recommendation to cite specific previously published works, please review and evaluate these publications to determine whether they are relevant and should be cited. There is no requirement to cite these works unless the editor has indicated otherwise.

**(Response)** There were no recommendations to add specific previously published works.

2. Please review your reference list to ensure that it is complete and correct. If you have cited papers that have been retracted, please include the rationale for doing so in the manuscript text, or remove these references and replace them with relevant current references. Any changes to the reference list should be mentioned in the rebuttal letter that accompanies your revised manuscript. If you need to cite a retracted article, indicate the article’s retracted status in the References list and also include a citation and full reference for the retraction notice.

**(Response)** We have checked that there are no errors in the list of references. In the twelfth line of the second paragraph of the Introduction, the reference to hypertension has been partially amended. (The citation for reference 35 has been removed.)

**RESPONSE TO REVIEWER #2**

**(Comment)**

Thank you for carefully addressing all comments; the manuscript has improved substantially and all requested points have been satisfactorily met. As a final minor editorial correction, please ensure consistent statistical terminology by replacing “no positive association” with “no significant association” in the opening sentence of the Discussion and in the Conclusion, to align with the Abstract and the reported statistical results.

**(Response)**

Thank you for pointing that out. We revised them as you mentioned.
